# State of the Science: Health Care Provider Communication of Cannabis Use Among Adults Living with Cancer

**DOI:** 10.1007/s13187-024-02484-z

**Published:** 2024-08-19

**Authors:** Amrit Baral, Bria-Necole A. Diggs, Judith Greengold, Cynthia Foronda, Debbie Anglade, Marlene Camacho-Rivera, Jessica Y. Islam, Denise C. Vidot

**Affiliations:** 1 Division of Epidemiology, Department of Public Health Sciences, University of Miami, 1120 NW 14th Street, Miami, FL 33136 USA; 2 Sylvester Comprehensive Cancer Center, Miami, FL 33136 USA; 3 School of Nursing and Health Studies, University of Miami, Coral Gables, FL 33146 USA; 4 SUNY Downstate Health Sciences University, Brooklyn, NY 11203 USA; 5 Moffitt Cancer Center, Tampa, FL 33612 USA

**Keywords:** Cannabis, Cancer, Communication, Palliative care

## Abstract

Despite medicinal cannabis gaining popularity for managing symptoms in cancer patients, a knowledge gap exists in patient-provider communication crucial for monitoring outcomes, optimizing dosing, and educating healthcare providers to integrate cannabis into treatment plans. Our goal is to understand communication dynamics, identify gaps, and pave the way for effective cannabis communication for individuals living with cancer (PLWC). We searched PubMed, CINAHL, and EBSCO for articles published between 2013 and July 2023, capturing the key concepts of cannabis use in cancer patients and their communication with healthcare providers in oncology settings. Preferred Reporting Items for Systematic Reviews and Meta-Analyses (PRISMA) statement guided the review. Studies were appraised by applying the Johns Hopkins Evidence-Based Practice Model for Nursing and Healthcare Professionals. Of the 2384 articles reviewed, 14 met the inclusion criteria. Three were qualitative studies, and 11 were cross-sectional surveys. All studies were level III evidence. Studies captured patients’ and providers’ perspectives; five were conducted among cancer patients, and nine were among healthcare providers in oncology settings. Findings revealed variations in healthcare provider recommendations, patient-initiated discussions, and barriers to discussing medical cannabis (MC). The synthesis of this evidence highlights the complexities surrounding MC in oncology settings, including knowledge gaps among healthcare providers, patient-initiated discussions, and challenges in accessing and prescribing medicinal cannabis. This review contributes valuable insights into the current landscape of MC use in cancer care, emphasizing the need for improved communication, education, and support for both patients and healthcare providers.

## Introduction

Cannabis use for medicinal purposes has become popular in recent years, especially for symptom or treatment effects management among people living with cancer (PLWC) [1]. Prior research studies have well-documented the use of cannabis for the management of various symptoms including nausea, vomiting, and inducing sleep and appetite in PLWC [2, 3]. With the current trend of cannabis legalization and normalization in the United States (US) and overseas, its use is increasing rapidly among the general population [4]. In the US, medical cannabis (MC) is now legally permitted in 38 states, four US territories, and the federal District of Columbia (D.C.). Furthermore, non-medical use of cannabis has been legalized in 23 states, three US territories, and D.C. [5, 6]. The prevalence of cannabis consumption is notably higher in North America, as well as affluent European and Oceania nations, in comparison to low and middle-income countries where there has been a smaller but rising trend in cannabis use [7]. Despite varying cultural practices, cancer is an internationally accepted medical condition that qualifies for the clinical application of cannabis; PLWC frequently use cannabis as a means of symptom and treatment side-effect management [6, 8]. Recent surveys reveal that 25% to 40% of cancer patients use cannabis, obtained from state-regulated dispensaries or illicit sources, primarily to manage symptoms like pain and anxiety [9].

PLWC often face heightened stress and anxiety due to their diagnosis and treatment. Cannabis is commonly used for symptom palliation, with approximately 40% of patients in the US, Canada, and Israel using it [10–12]. However, PLWC frequently lack guidance from their clinicians regarding appropriate dosages, consumption frequency, and specific product usage [13]. Clear communication regarding cannabis use between PLWC and healthcare providers is crucial for monitoring therapy outcomes, optimizing dosing amid potential drug interactions, reducing the necessity for external consultations, and educating healthcare providers to seamlessly integrate cannabis into existing treatment plans [14]. Despite the increase in legalization and normalization of cannabis, studies reveal a significant knowledge gap among healthcare professionals including understanding of cannabis-related recommendations for patients and evolving regulatory guidelines [15]. Healthcare providers frequently experience a lack of adequate knowledge regarding the use of cannabis by PLWC. Additionally, healthcare providers, including oncologists, face challenges in staying up to date with cannabis science research and providing effective communication to their patients due to their lack of training around MC usage [16, 17]. On the other hand, PLWC face challenges in initiating conversations with their providers regarding cannabis use because of the stigma attached to it [11]. In a survey of 1,592 individuals treated at a National Cancer Institute-designated Comprehensive Cancer Center, it was observed that although approximately one-third of cancer patients engaged in discussions about MC with their healthcare providers, a smaller fraction received actual recommendations or instructions on how to utilize MC from their healthcare providers [18].

Along with the increased use of cannabis among PLWC, there is a dearth of knowledge around patient-provider communication in this realm, suggesting a need for a comprehensive compilation of available evidence and identification of gaps. To our knowledge, this is the first comprehensive synthesis of the available evidence regarding patient-provider communication around cannabis use in oncology settings. The purpose of this systematic review is to understand the dynamics of patient-provider communication surrounding MC use in cancer care and identify urgent gaps to pave the way for effective communication on cannabis use for PLWC.

This systematic review aims to:

1. Assess how often patients disclose MC use to healthcare providers and the extent of medical advice given in cancer care.
2. Analyze healthcare providers' knowledge, beliefs, and practices regarding MC, including their comfort in making recommendations and discussing it with patients.
3. Examine factors affecting providers' reluctance to discuss MC, including the role of clinical evidence.
4. Identify barriers and information needs related to recommending and monitoring MC use in cancer patients.
5. Determine the training and information needs of healthcare providers on MC in oncology settings.

## Methods

### Overview

Healthcare providers’ communication of cannabis use among PLWC was explored using guidelines for reporting systematic reviews. The study team followed the Preferred Reporting Items for Systematic Reviews and Meta-Analyses (PRISMA) statement to complete this report [19]. No pre-registration was performed for this systematic review. The screening process as well as the management of collected articles were conducted using the Covidence website (www.covidence.org). We chose a systematic review approach to provide a comprehensive and unbiased synthesis of the limited existing evidence on this topic. This methodology allowed us to uncover international evidence to evaluate the quality and findings of included studies, ensuring that our conclusions are based on high-level evidence to inform clinical practice and future research.

### Eligibility Criteria

Articles were included in this systematic review if they were formally published research studies. The study population included PLWC and healthcare providers working in oncology settings. To ensure a comprehensive approach, the inclusion criteria were deliberately broad, aiming to encompass a wide range of relevant articles. The inclusion criteria for this review included (1) articles published from 2013- 2023, (2) studies related to humans, (3) published research studies, and (4) articles published in English. Exclusion criteria included: 1) articles published prior to 2013, (2) studies not related to humans, (3) review papers, experts' opinions, case reports, letters, or editorials and unpublished manuscripts, and (4) articles published in a language other than English.

### Information Sources and Search Strategy

An experienced team of researchers developed comprehensive search strategies for three electronic scientific databases—PubMed, CINAHL, and EBSCO—on July 11–12, 2023. We used keywords and MeSH terms based on the Population, Intervention, Comparison, Outcomes, and Study Design (PICOS) Model to search for articles on PLWC and provider communication regarding cannabis use in oncology settings. The articles were searched using the keywords and MeSH terms “Cannabis,” “Information,” and “Cancer,” combined with the Boolean operators “AND” and “OR” to capture the study’s key concepts: cannabis use in cancer patients and their communication to healthcare providers in oncology settings. The detailed list of search strategies employed is depicted in Table 1.

**Table 1:** The Search Strategy of the Research

Search term(s)	Search String
Cannabis	cannab* AND/OR marij* AND/OR hemp* AND/OR hashish AND/OR indica AND/OR sativa AND/OR CBD AND/OR Ganja* AND/OR Bhang*
Communication	communic* AND/OR educ* AND/OR inform* AND/OR misinform* AND/OR miscommunic*
Final combined string: (cannab* AND/OR marij* AND/OR hemp* AND/OR hashish AND/OR indica AND/OR sattiva AND/OR CBD AND/OR Ganja* AND/OR Bhang*) AND (communic* AND/OR educ* AND/OR inform* AND/OR misinform* AND/OR miscommunic*) AND (cancer AND/OR oncolog* AND/OR neoplas*)	

### Data Extraction and Quality Assessment

After retrieving articles from the three databases, three reviewers (AB, BD, and JG) independently screened titles and abstracts for relevance. Next, full texts of the remaining articles were examined and analyzed to ascertain their eligibility for inclusion. Disagreements were resolved through consensus. Articles were included if they reported data on PLWC and provider communication regarding cannabis use in oncology settings. Quality assessment of included articles was performed using the Johns Hopkins Evidence-Based Practice Model for Nursing and Healthcare Professionals [20]. This model grades studies based on study design on a scale from I (higher level evidence) to III (lower level evidence). Methodological quality was reviewed independently by AB, BD, and CF, with disagreements resolved by discussion or a fourth author (DV). Data on authors, publication country, title, purpose, design, provider type, variables, and major findings were extracted for comparison (Table 2).

**Table 2:** Study Characteristics from Included Research Articles

Author(s)/reference, country	Study purpose & Aim	Research Design	Study sample (N), Characteristics, and Setting	Major Finding(s)	Quality of study and Limitations
Braun IM, et al. (2021). *Cancer patients' experiences with medicinal cannabis-related care* https://doi.org/10.1002/cncr.33202 Epub 2020 Sep 28 USA	To assess the degree of medical cannabis (MC)-related health care oversight, MC practices and key information sources	Qualitative Semi-structured interview guides	24 PLWC with state/district authorization to use medical cannabis from 7 states throughout the US (median age, 57 years; range, 30‐71 years; 16 women [67%]),	Patients disclosed their MC use to their medical teams but received little medical advice about whether and how to use MC. Patients with cancer used MC for multipurpose symptoms management and as cancer-directed therapy, sometimes in lieu of standard of care treatments. Personal experimentation was an important source of MC know-how. Absent formal advice from medical professionals, patients relied on nonmedical sources for MC information 5 themes emerged: -Most participants received MC certifications through brief, perfunctory meetings with unfamiliar professionals -Patients disclosed MD use to their established medical teams but received little advice -Self-monitoring served as an important source of MC know-how -patients relied on Non-Medical and anecdotal sources for MC information -Patients with cancer used MC for multipurpose symptom management and cancer directed therapy	Level III (Johns Hopkins EBP Model for Nursing and Healthcare Professionals) Note: limitations included a convenience sample and lack of representation of Asians, Hispanics, and African Americans
Braun IM, et al. (2018) *Medical Oncologists' Beliefs, Practices, and Knowledge Regarding Marijuana Used Therapeutically: A Nationally Representative Survey Study* https://doi.org/10.1200/JCO.2017.76.1221 USA	To examine oncologists’ beliefs, knowledge, and practices regarding medical marijuana (MM)	Quantitative, cross-sectional, descriptive surveys a) Clinical discussions, recommendations, and knowledge; b) comparative effectiveness, and c) comparative risks; and d) predictors. The surveys included items from existing surveys as well as new items based on interviews from key oncology informants	237 medical oncologists across the US 65.8% were male, 57.9% were white, 36.0% completed oncology training ≥ 25 years ago, 52.8% held a medical school appointment, 52.8% practiced outside of a hospital setting, and 40.8% saw more than 60 patients per week	Only 30% of oncologists felt sufficiently informed to make recommendations regarding MM, 80% conducted discussions about MM with patients, and 46% recommended MM clinically. Sixty-seven percent viewed it as a helpful adjunct to standard pain management strategies, and 65% thought MM is equally or more effective than standard treatments for anorexia and cachexia. Findings identify a concerning discrepancy between oncologists’ self-reported knowledge base and their beliefs and practices regarding MM. Although 70% of oncologists do not feel equipped to make clinical recommendations regarding MM, the vast majority conduct discussions with patients about MM and nearly one-half do, in fact, recommend it clinically	Level III (Johns Hopkins EBP Model for Nursing and Healthcare Professionals) Note: Limitations included a small sample size and lack of generalizability to physicians in other specialties or practicing in other countries that have legalized MM on a federal level
Buchwald D; et al. (2022) *Perception of Patients with Cancer Enquiring About Adjuvant Therapy with Cannabis Medicine for Palliation of Symptoms: An Interview Study among Danish Health Care Professionals* DOIi: 10.1089/pmr.2021.0056 Denmark	The aim was to facilitate patient access to adjuvant therapy using medicinal cannabis under the guidance of physicians	Qualitative descriptive design using thematic analysis Semi-structured interview guides; Interviews were conducted in five groups with 10 health care providers in each group	50 Health Care Professionals (oncologists, palliative care specialists, general practitioners, registered nurses in oncology, and in palliative care) from Denmark	The informants reported that optional CM as adjuvant therapy was only discussed when initiated by the patient or relatives. Reluctance by HCPs to enter into a dialogue about CM with their patients was mainly explained by the lack of clinical evidence for the use of CM in palliative care of patients with cancer. None of the oncologists had ever prescribed CM, while three palliative care specialists and two general practitioners had issued prescriptions on rare occasions Three themes emerged: -Dialogues, “*We tend to feel that if we reject a discussion about cannabis, we also reject the patient. At least, this is how we think the patient may feel.*” “*It is not something we talk about as a routine unless the patient has raised liver enzymes.*” -Dilemmas, and “*We do not want to be a cannabis prescription factory with general practitioners referring cancer patients to us*.” -Prescription Practices “*To give cannabis as an indication and hope for curing cancer makes no sense to me as a physician.*” *I feel a great responsibility. If there were any side effects, I would be the one to blame.*” “*I do not prescribe cannabis. I would refer the patient to the palliative team.*”	Level III (Johns Hopkins EBP Model for Nursing and Healthcare Professionals) Note: Limitations include some interviews being collected by phone
Cousins, M., et al.. (2023) *Cannabis Use in Patients Seen in an Academic Radiation Oncology Department* USA	The aims were to assess patients’ willingness to discuss cannabis-related topics; estimate the prevalence of cannabis use among radiation oncology patients; characterize patient-reported motives, modes of administration and usage frequency; identify key areas where healthcare providers could potentially intervene, considering current understanding of the risks and benefits associated with cannabis	Quantitative, cross-sectional study; descriptive + analytical EMR tool i.e.The Michigan Radiation Oncology Analytics Resource (M-ROAR) was used to capture data from medical records History of cannabis use, reason for cannabis use, mode of administration, and frequency of use	Adult patients who agreed to answer questions related to cannabis use (*n* = 3052) at an academic radiation oncology department in Michigan seen for initial consultation or follow-up between October 2020 and November 2021 Recent cannabis use was reported by 11.2% of participants, while 7.7% were classified as non-recent users, and the majority (81.1%) had never used cannabis. Among those who reported recent cannabis use, 61.1% used it daily, with the remaining 17.7% using it less than weekly, 21.2% at least weekly but not daily, and 6.2% multiple times per day	Out of 3,143 patients, 91 (2.9%) chose not to answer cannabis-related questions *Univariate findings:* Patients over 50 were less likely to withhold answers (OR: 0.433; 95% CI: 0.272–0.718; *P* = .001) Patients with curative intent were less likely to decline to answer than those with palliative intent (OR: 0.580; 95% CI: 0.382–0.883; *P* = .011) Patients with prior radiation history were more likely to decline to answer (OR: 2.165; 95% CI: 1.416–3.310; P < 0.001) *Multivariable model:* Age, treatment intent, and prior radiation history remained significant	Level III (Johns Hopkins EBP Model for Nursing and Healthcare Professionals) Limitations: - Self- reported data subject to recall or social desirability - Single center study limiting generalizability
Filetti M., et al. (2021) *Knowledge and attitudes of Italian medical oncologists and palliative care physicians toward medical use of cannabis in cancer care: a national survey* https://doi.org/10.1007/s00520-021-06383-7 Italy	The aim of this study was to assess the knowledge and attitude of Italian cancer care professionals toward medical cannabis prescription	Quantitative, Cross-sectional study design Web-based survey sent electronically via email between 1 May and 30 September 2019 Variables collected inlcuded: age, sex, medical speciality, workplace and work region, are of interest (type of malignancy treated), and patient population (adult/pediatric)	Associates of the Italian Association of Medical Oncology, and Italian Association of Palliative care (N = 475) who replied to the questionnaire. 46% Male, 71% > 40 years old, 61% medical oncology/hematology as specialization. Regarding the patient population 92% were adults	90% of respondents reported familiarity with medical cannabis, with 79% being approached by patients or caregivers (66%) about it. Approximately half of the respondents received requests for medical cannabis prescriptions from patients (57%) or caregivers (45%), but only 29% had actually prescribed it to cancer patients. The most commonly prescribed formulation in Italy was cannabis FM2. The primary clinical indications for medical cannabis use were pain, gastrointestinal issues, and mood disorders. Only 9 respondents reported side effects such as anxiety, insomnia, and muscle spasms. When asked about the normative references for medical cannabis prescription and use in Italy, only 14% were able to mention the specific legislative reference	Level III (Johns Hopkins EBP Model for Nursing and Healthcare Professionals) Limitations: Low response rate, included doctors of specialized medical societies only
Hewa-Gamage D., et al. (2019) *A Cross-sectional Survey of Health Professionals' Attitudes toward Medicinal Cannabis Use as Part of Cancer Management* Australia	The aim of the study was to evaluate the attitudes of health professionals toward the use of medicinal cannabis as part of the management of patients with cancer	Quantitative Cross-sectional survey that included a qualitative question	Health professionals aged 18 or older (N = 135) recruited from a public, metropolitan hospital in Australia which is dedicated to the management of patients with cancer, and cancer related research. Sixty-six participants were medical and 66 were non-medical practitioners, which included 32 nurses, 10 pharmacists and 24 allied health workers and other disciplines. 63% were female	84% of participants were aware of the legislative changes made in Victoria regarding medicinal cannabis. Only 11% of health professionals felt sufficiently informed about the use of medicinal cannabis under current legislation. 29% reported sufficient knowledge of the evidence base for medicinal use of cannabis in cancer. 20% were aware of potential drug interactions. 62% reported that their patients inquired about medicinal cannabis. None stated that they prescribed medical cannabis to cancer patients. 34% would recommend medicinal cannabis to their patients with cancer, 20% would not, and 46% were unsure. Comments indicated concerns about lack of clinician knowledge, drug efficacy, side effects and drug interactions	Level III (Johns Hopkins EBP Model for Nursing and Healthcare Professionals) Limitations: Small sample size limiting generalizability, low response rate (13%), possibility of filling out surveys multiple times
McLennan, A., et al.. (2020). *Health care provider preferences for, and barriers to, cannabis use in cancer care* https://doi.org/10.3747/co.27.5615 Canada	The aim of the study was to assess the knowledge, beliefs, barriers, and preferences regarding medical cannabis among oncology healthcare practitioners in Canada	Quantitative, descriptive, Cross-sectional survey	Healthcare providers (N = 103) employed by Alberta Health Services, Cancer Control Alberta working with cancer patients directly or indirectly	75% of respondents were women. The majority of participants were oncology nurses (40%), radiation therapists (9%), and pharmacists (6%). 75% of respondents provided direct care to cancer patients. A significant portion (69%) had discussed cannabis with a patient in the last month, while 84% felt they lacked adequate cannabis knowledge for recommendations. Commonly cited barriers included monitoring cannabis use (54%), prescribing accurate doses (61%), selecting the right strain (53%), and limited research (50%). Over half of healthcare professionals (53%) expressed interest in additional information or training regarding cannabis use in oncology	Level III (Johns Hopkins EBP Model for Nursing and Healthcare Professionals) Limitations: Underrepresentation of other providers, based on a single medical center, low response rate
Oldfield K, et al. (2022) *Experiences, patient interactions and knowledge regarding the use of cannabis as a medicine in a cohort of New Zealand doctors in an oncology setting* https://doi.org/10.1136/postgradmedj-2020-139013 New Zealand	To explore the experiences, patient interactions and knowledge regarding the use of cannabis as a medicine in New Zealand doctors in an oncology setting November 2019 to January 2020	cross-sectional survey Variables included: 1) Doctor-patient interactions relating to the use of cannabis as a medicine, either prescribed or illicit. 2) Facilitation of prescriptions of cannabis-based products and impediments to the process. 3) Knowledge of conditions with evidence for and against the use of cannabis-based products. 4) Knowledge of the regulatory processes in place for cannabis-based products in NZ; approval, funding and importation. 5) Knowledge of pharmaceutical cannabis-based products; name, constituents, availability in NZ, formulation, and cost to patients. 6) Previous education regarding the use of cannabis-based products and preference for future education resources	Health care providers (N = 45) at a secondary-care hospital oncology departments in New Zealand	- 84% of doctors had received requests from their patients for cannabis-based products—98% of doctors reported to having patients request a prescription for cannabis-based products—73% of doctors cited knowledge of at least one cannabis based product—82% expressed future prescribing concerns but also showed willingness to use cannabis based product	Level III (Johns Hopkins EBP Model for Nursing and Healthcare Professionals) Limitations: sample consists of doctors that attend CME sessions, limiting generalizability—study design could lead to self-report bias due to self administering of surveys
Panozzo S, et al. (2020) *Who is asking about medicinal cannabis in palliative care?* https://doi.org/10.1111/imj.14732 Australia	To characterize the population of patients and/or carers who initiate discussions about and requests for medicinal cannabis and the clinical outcomes of these discussions	cross-sectional study	*N* = 104 Data was prospectively collected from 3 major metropolitan teaching hospitals in Victoria, Australia from 2018 to 2019	- 93% of discussions were initiated by the patients and/or carers - 25% of discussions pertained to patients or carers inquiring about medical cannabis as a form of cancer control or cure - 30 patients reported self-medicating with cannabis - Of the patients self-medicating with cannabis, over half used via cannabis oil - 27% of discussions resulted in either medical cannabis being prescribed, or an existing medical cannabis prescription managed or discussed	Level III (Johns Hopkins EBP Model for Nursing and Healthcare Professionals) Limitations: process followed for data collection did not allow for detailing examination of individual patients - underreporting may have occurred, as some providers may not have disclosed all discussion that were had
Patell R, et al. (2022). *Oncology Fellows' Clinical Discussions, Perceived Knowledge, and Formal Training Regarding Medical Cannabis Use: A National Survey Study* https://doi.org/10.1200/OP.21.00714 USA	To determine whether oncology training adequately prepares fellows to discuss medical cannabis	Cross-sectional study survey assessing frequency of clinical discussions and nature of initials for medical cannabis - perceived knowledge - perceived effectiveness compared to conventional care - perceived risk compared to opioids for mood disorders - prior training in medical cannabis and sources of information	Oncology fellow (*N* = 189) January 2021 to March 2021 electronic survey distributed to training programs across the United States	- 40% response rate of trainees that completed the surveys - only 13% of trainees felt sufficiently informed in order to recommend cannabis - less than ½ receive formal training (24%) - fellows that had previously had training were more likely to discuss cannabis with patients (p = 0.002) - More than 40% of trainees found cannabis to be useful adjunctive therapy for anorexia, nausea, and pain	Level III (Johns Hopkins EBP Model for Nursing and Healthcare Professionals) Limitations: self-report bias from online survey design - the response rate was less than 50%
Zylla, et al. [53] *Oncology Clinicians and the Minnesota Medical Cannabis Program: A Survey on Medical Cannabis Practice Patterns, Barriers to Enrollment, and Educational Needs* https://doi.org/10.1089/can.2018.0029 USA	To delineate oncology providers’ views on medical cannabis, identify barriers to patient enrollment, and assess clinicians’ interest in a clinical trial of medical cannabis in patients with stage IV cancer	Cross-sectional 14-item survey to identify providers’ practices, knowledge, and attitudes about medical cannabis as well as to assess barriers to certifying patients in the Minnesota Medical Cannabis Program (MMCP)	Physicians and NPs in hematology/oncology (*N* = 153) June to August of 2017 Study survey was sent via mail and e-mail to providers identifying as hematology and/or oncology specialty in the Minnesota Board of Medical Practice database and oncology Nurse Practitioners database	- 65% of respondents supported the use of medical cannabis - higher barrier to MMCP patient enrollment identified was perceived cost and inadequate research - 36% lacked confidence in discussing the risks and benefits of medical cannabis - 85% reported wanting more education on medical cannabis	Level III (Johns Hopkins EBP Model for Nursing and Healthcare Professionals) Limitations: Small sample size with limited generalizability - Low response rate compared to other studies - Self-selected survey enrollment could lead response bias
Black KA, et al. (2023) *Cannabis use in gynecologic cancer patients in a Canadian cancer center* Canada	To determine the frequency of cannabis consumption among gynecologic cancer patients and elucidate the nature of their cannabis usage. Investigate the primary sources from which patients gather information about cannabis. Provide an overview of the cannabis consumption trends within the gynecologic cancer patient population	Quantitative, Cross-sectional electronic survey	Gynecologic cancer patients 18 years and older (*N* = 46) attending gynecologic clinic and receiving treatment at the Tom Baker Cancer Center in Calgary, Alberta, Canada	-Most common cancer sites were ovarian (50%) and uterine (32.6%) -Majority were receiving chemotherapy (45.6%) -37% were current cannabis users -Most common symptoms managed were pain, anxiety and insomnia -Most patients did not have prescription and obtained cannabis from recreational dispensary -Many did not talk to their doctors -Most common sources were cannabis retailers, friends/family -More than 50% of participants showed interest in discussing cannabis if their physician started the subject - 41.3% felt if their physician would be able to provide them information then that would help - 10.9% report it would help if their providers talk to them without judgment - Out of the 17 patients currently using cannabis, only 8 had discussed their cannabis use with their oncologist, and just 2 of those 8 believed their doctor adequately addressed their cannabis-related inquiries	Level III (Johns Hopkins EBP Model for Nursing and Healthcare Professionals) Limitations: - Small sample size - Single center study -Social desirability bias -lack of temporality i.e. whether cannabis use preceded cancer diagnosis -Survey in English limited recruitment of participants unable to read and write in English
Weiss MC, et al., (2022) *Cannabis Survey Study of breast cancer patients' use of cannabis before, during, and after treatment* USA	To describe the patterns of cannabis consumption among breast cancer patients. This includes understanding why and when they use cannabis, where they obtain information and products related to cannabis, how satisfied they are with the information they receive, their perceptions of the safety of cannabis, and their conversations with their doctors about cannabis	Quantitative Cross-sectional electronic survey design	Individuals (*N* = 612), (18 years and older with reported a diagnosis of breast cancer within the past five years) in the United States who were part of the Breastcancer.org community, a nonprofit online platform offering medical guidance and peer support	- Mean age was 57 years with 605 females, 5 males, and 2 that preferred not to answer - 42% reported using cannabis to address medical issues - Of those who use cannabis to address medical issues, 23% use it for medical purposes stemming from their illness or its treatments - Most were unsatisfied with the information sought on cannabis use for medical purposes - 39% discussed cannabis with any of their physicians - 76% of discussions were patient initiated - Older patients were more likely to ask their physician about cannabis - 28% of all respondents were uncomfortable discussing cannabis with their providers - Among those who discussed younger patients were more likely to feel their providers were supportive - Among the 612 participants, 42% (257 individuals) used cannabis to alleviate symptoms such as pain (78%), insomnia (70%), anxiety (57%), stress (51%), and nausea/vomiting (46%) - 49% of cannabis users believed in the potential of medical cannabis for cancer treatment. Among those using cannabis, 79% had used it during various treatments, including systemic therapies, radiation, and surgery	Level III (Johns Hopkins EBP Model for Nursing and Healthcare Professionals) Limitations: - Selection biases may favor cannabis users, patients with advanced disease, and residents in cannabis-legal states -Underreporting of cannabis use is due to its illegal status and social stigma -Recall bias may cause incomplete data on sources and patterns of use, blurring the line between medical and recreational use -Limited access to the online, English-only survey
Wilson A & Davis C. (2022). *Attitudes of cancer patients to medicinal cannabis use: a qualitative study* Australia	To explore the attitudes, barriers, and concerns of cancer patients from one regional community in Australia to gain deeper insights into their experiences faced in using medicinal cannabis	Qualitative study utilizing focus groups (both online and in person) or one-on-one interviews	Cancer patients, English speaking, 18 years and older, interested in medical cannabis (*n* = 16) from one regional community in Australia	Perceived Benefits - Participants reported substantial physical and psychological benefits, particularly in addressing common cancer-related symptoms - Medicinal cannabis improved chemotherapy tolerance and emotional well-being - Cannabidiol (CBD) oils help manage symptoms and emotional challenges - Despite initial concerns, most participants found medicinal cannabis safe and effective Access Difficulties - Access issues were prevalent, with difficulties in securing legal prescriptions and challenges related to the approval process - The illegal status complicates disclosure to healthcare professionals - Information on obtaining medicinal cannabis was elusive - The high cost of medicinal cannabis was a financial barrier Uncertainty -Frustration stemmed from a lack of conclusive evidence, limited GP knowledge, and misunderstandings about medicinal cannabis -Some participants believed it could treat cancer directly -Participants sought information from non-medical sources, highlighting the need for accessible, reliable information Support - Positive support from family and friends, with minimal stigma -Cancer support groups offered camaraderie and information -Mixed experiences with GPs, some of whom were supportive	Level III (Johns Hopkins EBP Model for Nursing and Healthcare Professionals) Limitations: -Recruited participants from only one regional area resulting in a small sample - Selection bias from self-selection/volunteers -Non-representation of non-English speakers - Combination of online and in-person formats for conducting the focus groups, which could have influenced how group dynamics operated and how participants engaged

## Results

**Fig. 1:**
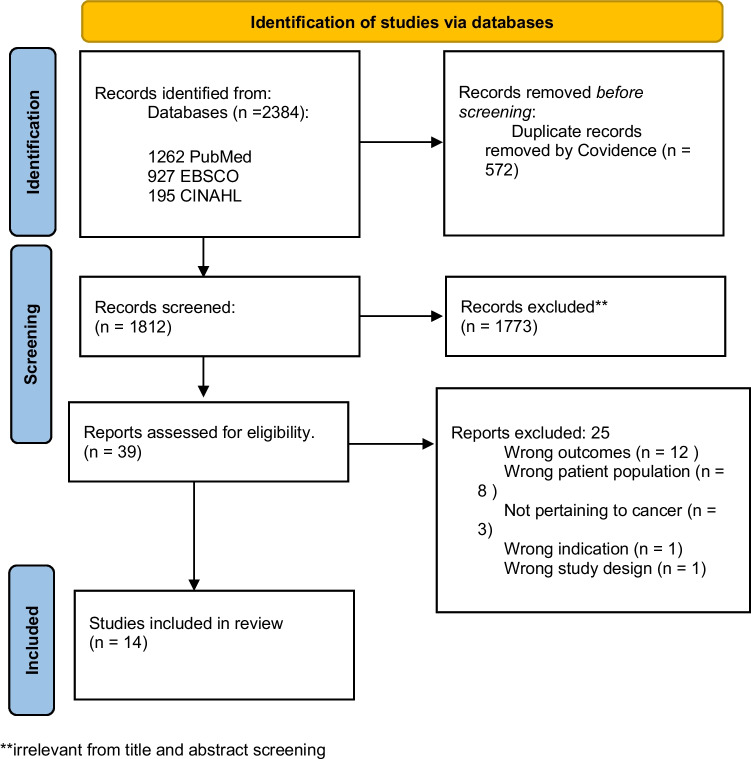
The process of screening articles based on the PRISMA 2020 flow diagram

The flow diagram of the study selection process is provided in Fig. 1.

Of the 14 studies included, three were qualitative with sample sizes of 16 to 50 [21–23]. Whereas 11 were cross-sectional with sample sizes of 45 to 3,052; one cross-sectional study used a mixed method [15, 24–33] **(**Table 2**)**. According to the Johns Hopkins Evidence-Based Practice Model for Nursing and Healthcare Professionals, all studies were level III evidence. Studies captured perspectives from both patients and providers: five among cancer patients and nine among oncology healthcare providers. Five studies were conducted in the United States, with the rest in Denmark (n = 1), Italy (n = 1), Australia (n = 3), Canada (n = 3), and New Zealand (n = 1) (Table 2).

The cross-sectional studies aimed to gather quantitative data on various aspects of MC, including patient behaviors and attitudes, such as willingness to discuss cannabis; the prevalence of use among PLWC and motives for usage; healthcare professionals' knowledge and attitudes in primary care and oncology settings; oncology fellows' preparedness to discuss MC; and the frequency and nature of cannabis consumption and information sources. The qualitative studies aimed to explore in-depth aspects of MC, including healthcare oversight and practices, oncologists' beliefs and practices, and patient access to adjuvant therapy with physician guidance. They also examined patient interactions and knowledge about medicinal cannabis. Through narratives and thematic analysis, the studies uncovered cancer patients' attitudes, barriers, and concerns, offering a comprehensive view of their experiences with MC.

### Studies Reflecting Providers’ Perspectives

In a 2018 US survey conducted by Braun et al., among 400 medical oncologists, 79.8% discussed MC with patients, and 45.9% recommended it for cancer-related issues in the past year. The discussion rate was higher in the West (94.7%) compared to the South (68.9%), with recommendation rates of 84.2% in the West and 34.7% in the South. Oncologists seeing more than 60 patients per week were more likely to discuss MC. Among those discussing MC, 78% had patient-initiated conversations. Only 29.4% felt knowledgeable enough to make recommendations, and 56.2% of those recommending MC lacked confidence in their knowledge. While lacking consensus on MC as a primary pain treatment, over two-thirds supported its use as an adjunct to standard pain management [24].

In a 2021 Italian study by Filetti et al., an electronic questionnaire assessed cancer care professionals' knowledge and attitudes toward MC prescription. Out of 2,616 respondents, 475 (18%) were analyzed. Most were medical oncologists/hematologists (61%) and anesthesiologists/pain specialists (13%) focusing on breast (20%), gastrointestinal (29%), and lung cancers (26%). Most were familiar with MC (90%), having been asked about it by patients (79%) or caregivers (66%). Approximately half of the healthcare professionals reported receiving requests to prescribe MC, but only 29% had actually done so. Few cited legislative references, and there was a notable lack of knowledge regarding the comparative effectiveness and safety profile of MC. Factors increasing the likelihood of prescribing included the professional's age, patient requests, positive views on effectiveness, and familiarity with normative references [26].

In a 2019 survey at an Australian cancer hospital, Hewa-Gamage et al. evaluated the attitudes of 150 health professionals (32 nurses, 10 pharmacists, and 24 allied health workers) toward medicinal cannabis in cancer care. Sixty-two percent of the respondents reported patient inquiries about medicinal cannabis, but over half felt insufficiently informed about access (74%), evidence base (59%), and potential drug interactions (65%). Recommendations varied, with 34% in favor, 20% against, and 46% unsure. Despite awareness of legislative changes in Victoria, only 11% felt informed about accessing medicinal cannabis. Twenty-nine percent were knowledgeable about its evidence base and 20% about drug interactions. While 77% of medical practitioners would "consider" prescribing it, none had done so. Education and knowledge gaps were common concerns, with 62% unsure about recommending medicinal cannabis despite receiving patient inquiries [27].

In a 2020 study, McLennan et al. surveyed 103 oncology healthcare providers (HCPs) at the Tom Baker Cancer Center in Calgary to identify barriers and preferences regarding MC. Most respondents were female (75%), including oncology nurses (40%), radiation therapists (9%), and pharmacists (6%). About 75% were directly involved in patient care. Findings revealed that 69% had discussed cannabis with a patient in the past month, and 84% felt insufficiently informed. Barriers included monitoring patient use (54%), prescribing accurate doses (61%), and lack of research (50%). Fifty-three percent wanted more training on cannabis in oncology. Notably, 69% had patients disclose cannabis use, and 41% were asked for advice. Additionally, 59% referred patients to other HCPs, mainly oncologists (35%) and MC practitioners (33%). Twenty-three percent requested patients stop using cannabis, citing concerns about interactions, harm, or lack of efficacy. Despite limited knowledge, many HCPs engaged with patients on cannabis and expressed interest in professional training on its use in oncology [15].

Oldfield et al. (2020) surveyed 45 doctors in New Zealand oncology settings to explore experiences with cannabis as medicine. Findings revealed that 37% received patient requests for cannabis-based products, and 98% noted patients using illicit cannabis. About 84% had patients who requested cannabis prescriptions, primarily for pain, cancer, and nausea. Among consultants, 46% attempted prescriptions, and 25% of registrars attempted. Reasons for not prescribing included a lack of knowledge around evidence and understanding. Despite concerns, 52% expressed a likelihood to prescribe a pharmaceutical-grade cannabis product. Side effects reported included drowsiness, nausea, and psychotic symptoms, with 73% aware of pharmaceutical-grade cannabis products. Educational sources included CME sessions, journals, Ministry of Health website, and others. CME sessions were preferred for future education. Some doctors faced impediments, like cost and paperwork in prescribing [28].

Panozzo et al. (2019) conducted a study within palliative care settings to understand information-seeking and requests for medicinal cannabis in clinician-patient consultations. The aim was to characterize patients and carers initiating discussions and the clinical outcomes. Twenty-eight palliative care clinicians in three major hospitals in Victoria, Australia, completed case reports for 104 interactions discussing medicinal cannabis. Patients and carers initiated 93% of discussions, with 66% indicating multiple reasons for cannabis use, primarily for pain, nausea, and poor appetite. One in four sought cannabis for cancer control or cure. About 40% of discussions involved requests for medicinal cannabis, and 27% resulted in prescription or management of existing ones [29].

Patell et al. (2022) surveyed 189 trainees from 40 US oncology fellowship programs, assessing MC practices and knowledge. Fifty-seven percent discussed cannabis with over five patients in the past year, but only 13% felt sufficiently informed to make recommendations. Formal training, received by 24%, was linked to increased discussions and confidence (risk ratio: 1.37, 5.06; P < 0.002, P < 0.001). Participants viewed cannabis as a useful adjunctive therapy, with 91% discussing it during training, often initiated by patients and families (80%). Common information sources were peer-reviewed literature (30%) and colleagues' lectures/webinars (29%). Formal training correlated with more frequent discussions (risk ratio: 1.48; P < 0.001). About 54% believed cannabis was somewhat more effective than conventional therapies for anorexia or cancer cachexia. Concerns included infections in immunocompromised patients using combusted MC. Identified barriers were lack of clinical data, training, stigma, and regulatory policies [30].

In 2017, Zylla et al. surveyed 552 oncology providers in Minnesota to assess their practices, knowledge, and attitudes toward MC. The study aimed to understand providers' views, identify barriers to patient enrollment, and assess interest in a MC clinical trial for stage IV cancer. Of 529 eligible participants, 153 (29%) responded, with 68 registered in the Minnesota Medical Cannabis Program. Most respondents were medical oncologists or medical oncology nurse practitioners/physician assistants (82%) with many practicing in the community setting (67%). For a patient scenario, 65% recommended MC, 25% opposed, and 10% skipped the question. Barriers included perceived cost, inadequate research, uncertainty about side effects/benefits, and lack of FDA approval. Additional concerns were abuse/misuse, quality of cannabis in Minnesota, legal ramifications for providers and patients, social stigma, and health group restrictions. About 36% lacked confidence in discussing risks/benefits, with registered respondents having greater confidence (12%). 85% desired additional training, preferring written summaries (75%), online programs (51%), symposiums/conferences (32%), and newsletters (18%) [31].

In a 2022 Danish study, Buchwald et al. conducted qualitative research with 50 healthcare professionals, including oncologists, palliative care specialists, general practitioners, and nurses, on their perceptions of cancer patients inquiring about MC for adjuvant palliative therapy. Professionals discussed MC only when initiated by patients or relatives due to a lack of clinical evidence in palliative care. Oncologists hesitated due to the absence of RCTs and inquired only if the patient showed adverse effects. Palliative care physicians engaged upon patient or caregiver requests. Nurses felt constrained by the lack of RCTs, and primary care physicians avoided the topic due to knowledge gaps. Oncologists viewed the Danish MC pilot program as politically driven, and the Danish Medicines Agency’s control frustrated primary care physicians. Oncology and palliative care nurses faced dilemmas and conflicting policies. Some palliative care physicians were willing to prescribe MC as a last resort, emphasizing respectful communication, while oncology physicians, understanding patients' hopes, refrained from prescribing on principle [22].

Studies on healthcare providers' perspectives on MC in oncology show common issues. Providers frequently get patient inquiries but feel inadequately informed to recommend MC. In the US and Canada, many oncologists discuss MC but lack confidence due to insufficient evidence and training. European and Australian providers also face barriers like limited clinical evidence and policy constraints. Overall, there's a clear need for better education and guidelines to help providers address MC inquiries effectively.

### Studies Focused on Patients’ Perspectives

Cousins et al. (2023) examined cannabis-related discussions among 3,143 adult radiation oncology patients in Michigan, using the Michigan Radiation Oncology Analytics Resource (M-ROAR) to capture medical record data on cannabis use, motives, administration modes, and frequency. Among patients, 2.9% (91 individuals) chose not to answer cannabis-related questions. Univariate findings indicated that patients over 50 were less likely to withhold answers as compared to younger patients (OR: 0.433; 95% CI: 0.272–0.718; P = 0.001), while those with curative intent were more open than those with palliative intent (OR: 0.580; 95% CI: 0.382–0.883; P = 0.011). Patients with prior radiation history were more likely to decline answers than those without radiation history (OR: 2.165; 95% CI: 1.416–3.310; P < 0.001). In the multivariable model, age (aOR:0.419; 95% CI: 0.251–0.700), treatment intent (aOR:0.539; 95% CI: 0.343–0.846), and prior radiation history (aOR:1.944; 95% CI: 1.222–3.092) remained significant factors influencing patients' willingness to discuss cannabis-related topics [25].

Weiss et al. (2021) studied cannabis use among US breast cancer patients, focusing on reasons, timing, information sources, satisfaction, safety perceptions, and physician discussions. Of 612 participants (age ≥ 18 years) from Breastcancer.org and Healthline.com communities, 42% used cannabis; 23% used it solely for medical reasons, while 77% used it both medically and recreationally. Sixty-four percent were very or extremely interested in MC. Among 302 seeking information, most were dissatisfied: only 6% found it extremely satisfying, while 25% were very satisfied, 44% somewhat satisfied, 19% minimally satisfied, and 6% dissatisfied. Thirty-nine percent discussed cannabis with their physicians, with 76% of these conversations initiated by patients. Older participants (≥ 66 years) were more likely to consult their physicians (87%) compared to younger groups (76% for ages 50–65 and 69% for < 50 years, respectively, p = 0.03). However, a notable 28% felt uncomfortable discussing cannabis with their physicians. Younger patients were more likely to view their physicians as supportive, with 72% of those < 50 years feeling very or extremely supported, compared to 52% in the 50–65 age group and 46% in the ≥ 66 years group, respectively (p = 0.03) [33].

Black et al. (2023) studied 46 gynecologic cancer patients, primarily with ovarian and uterine cancer, undergoing chemotherapy at Calgary's Tom Baker Center to assess cannabis use patterns. Among participants, 37% used cannabis, mainly for pain, anxiety, and insomnia, sourcing it from recreational dispensaries without prescriptions. About 50% hadn't discussed cannabis with their doctor, relying on retailers and friends/family for information. Over half were open to discussing cannabis if initiated by their physician. Non-users preferred friends/family and dispensaries for information. Only 8 of 17 current users had spoken to their oncologist about cannabis, and just 2 found their doctor helpful. Overall, 28.3% wanted to discuss cannabis with their doctor, while 56.5% would if the physician started the conversation, 41.3% if they provided useful information, and 10.9% if the discussion was non-judgmental [32].

In a 2021 Braun et al. study, 24 individuals in MC authorized states reported using it for symptom management and as a substitute for standard treatments, claiming antineoplastic properties. They self-experimented without formal medical advice, relying on non-medical sources. Most obtained MC certifications through brief meetings with unfamiliar professionals with limited access to quality clinical information and brief consultations. Discussions about MC were patient-initiated, and few received certifications from primary care providers or specialists. Many providers, though neutral, were reluctant to advise on MC [21].

Davis & Wilson (2022) conducted a qualitative study with 16 Australian cancer patients to explore attitudes and concerns about medicinal cannabis. Participants aged 36–82 experienced pain, poor sleep, nausea, anxiety, and depression from various cancer treatments. Due to COVID-19, they joined face-to-face or online focus groups and interviews. Common issues included sleep and pain concerns. Support from family and cancer support groups was generally positive, but some hesitated to discuss cannabis. Many did not disclose use to health professionals, citing systemic limitations and lack of GP support. They often turned to the internet or friends for information. Challenges included obtaining legal prescriptions and frustrations with GPs' limited knowledge about cannabis and its efficacy [23].

Studies on patients' perspectives on MC reveal that older patients are more likely to discuss it with healthcare providers, though there is dissatisfaction with the information and support received. Many patients rely on non-medical sources and are frustrated with limited professional guidance. Regardless of methods or sample sizes, a common finding is that patients often initiate cannabis discussions and lack comprehensive medical advice, highlighting the need for improved communication and education in oncology care.

## Discussion

Studies reveal a complex interplay of attitudes, knowledge gaps, and communication challenges in MC use. Providers show limited knowledge and confidence despite interest in prescribing MC. Patients increasingly view MC as a complementary therapy but face challenges due to insufficient physician support, systemic constraints, and reliance on non-medical sources. This discussion highlights key findings and their implications for the use of MC in cancer care.

### Limited Medical Guidance and Patient-Initiated Disclosures

Patients with cancer often initiate discussions and disclose their use of MC but receive minimal advice from their medical teams [21, 22, 29, 33]. Many clinicians feel insufficiently informed to make recommendations about MC, despite growing patient interest. This discrepancy between self-reported knowledge and clinical practice highlights a critical need for improved provider education and guidelines on MC.

### Patient-Driven Information and Self-Experimentation

The study highlights that patients often rely on personal experimentation and anecdotal sources for MC knowledge due to a lack of formal medical advice [21]. This underscores the need for comprehensive, evidence-based resources to help patients make informed decisions about cannabis use. The risks of self-experimentation, especially among medically fragile patients on complex treatments, are understudied but significant.

### Access and Prescription Practices

A key finding is the reluctance of healthcare providers, especially oncologists, to discuss MC due to a perceived lack of clinical evidence. None of the oncologist participants had prescribed cannabis, though some palliative care specialists and general practitioners had occasionally done so [22]. This suggests a potential role for specialists and underscores the need for more research and clinical evidence to guide prescription practices.

### Patient Characteristics and Disclosure Patterns

The study shows that patients over 50, those with curative intent, and those with a history of radiation are more likely to discuss MC use [25]. Recognizing these demographics helps providers approach discussions in a way that respects patient preferences and addresses concerns related to symptom palliation and prior radiation history.

### Healthcare Provider Perspectives and Barriers

The study findings emphasize that healthcare providers, including oncology nurses, radiation therapists, and pharmacists, encounter barriers in discussing MC. These barriers include a lack of knowledge, concerns about monitoring, dosing, strain selection, and limited research [15]. Providers clearly need more research, education, and training on MC to better assess treatment risks and benefits.

### Perceived Benefits and Access Difficulties

Participants reported potential benefits of MC, like improved chemotherapy tolerance and symptom management, underscoring its value in cancer care [23, 32]. However, access challenges—such as legal barriers, high costs, and lack of information—pose significant obstacles [23, 32]. Addressing these issues is crucial to ensure that patients who could benefit from MC can access it safely and legally, with proper monitoring by their oncology team.

### Uncertainty and the Need for Reliable Information

This review highlights PLWC’s frustration with the perceived lack of evidence, limited practitioner knowledge, and misunderstandings about MC [23]. Patients frequently turned to non-medical sources for information, emphasizing the need for accessible, evidence-based resources. While family, friends, and cancer support groups provide valuable support, mixed experiences with general practitioners highlight the need for standardized guidelines and provider education in this emerging field.

Significant gaps exist in education and communication about MC in cancer care. Patients often lack clinical guidance and rely on anecdotal sources [24]. Despite its potential to alleviate symptoms, federal illegality complicates patient-provider discussions [34]. Oncologists often feel unprepared to advise on MC, underscoring the need for enhanced education and motivational interviewing to improve dialogue [9]. Legislative changes like the States Reform Act may reduce stigma, but challenges such as a limited evidence base and societal stigma persist [34]. Cancer education professionals must engage in research and education to improve outcomes [35]. Continuing education for healthcare providers on MC is crucial, including workshops, seminars, online certification, case studies, peer-reviewed journals, and clinical trials [36]. Training in legal and ethical issues, combined with patient education, supports informed decision-making and helps navigate regulatory challenges [37].

## Limitations

The study designs and outcomes varied widely, complicating data extraction and synthesis. Quantitative studies lacked validated measures of cannabis knowledge and attitudes, hindering direct comparisons. With the topic still emerging, existing evidence levels were low. Despite this, the review mapped current knowledge and identified evidence gaps and research opportunities. Some studies might have been missed due to differing interpretations of selection criteria. To address this, we used Covidence software, established clear criteria, and held meetings to discuss the selection process. Non-English studies and those published before 2013 were excluded to focus on recent research.

## Conclusions

As interest in medicinal cannabis grows among people living with cancer, healthcare providers' knowledge, attitudes, and training on its use vary widely. With increasing legalization and cultural acceptance, it is crucial to invest in education for providers and promote non-judgmental, open communication with patients. Public health initiatives should also aim to de-stigmatize medicinal cannabis discussions among people living with cancer.

## Data Availability

All data supporting the findings of this research are available within the article or its tables.
